# Evaluation of a Metal Artifact Reduction Algorithm for Image Reconstruction on a Novel CBCT Platform

**DOI:** 10.1002/acm2.14516

**Published:** 2024-09-17

**Authors:** Abby Yashayaeva, Robert Lee MacDonald, James Robar, Amanda Cherpak

**Affiliations:** ^1^ Department of Physics and Atmospheric Sciences Dalhousie University Halifax Canada; ^2^ Department of Radiation Oncology Dalhousie University Halifax Canada; ^3^ Nova Scotia Health Halifax Canada

**Keywords:** cone beam CT, image quality, metal artifact reduction, quantitative image analysis

## Abstract

**Purpose:**

The presence of metal implants can produce artifacts and distort Hounsfield units (HU) in patient computed tomography (CT) images. The purpose of this work was to characterize a novel metal artifact reduction (MAR) algorithm for reconstruction of CBCT images obtained by the HyperSight imaging system.

**Methods:**

Three tissue‐equivalent phantoms were fitted with materials commonly used in medical applications. The first consisted of a variety of metal samples centered within a solid water block, the second was an Advanced Electron Density phantom with metal rods, and the third consisted of hip prostheses positioned within a water tank. CBCT images of all phantoms were acquired and reconstructed using the MAR and iCBCT Acuros algorithms on the HyperSight system. The signal‐to‐noise ratio (SNR), artifact index (AI), structural similarity index measure (SSIM), peak signal‐to‐noise ratio (PSNR), and mean‐square error (MSE) were computed to assess the image quality in comparison to artifact‐free reference images. The mean HU at various VOI positions around the cavity was calculated to evaluate the artifact dependence on distance and angle from the center of the cavity. The artifact volume of the phantom (excluding the cavity) was estimated by summing the volume of all voxels with HU values outside the 5th and 95th percentiles of the phantom CBCT with no artifact.

**Results:**

The SNR, AI, SSIM, PSNR, and MSE metrics demonstrated significantly higher similarity to baseline when using MAR compared to iCBCT Acuros for all high‐density materials, except for aluminum. Mean HU returned to expected solid water background at a shorter distance from metal sample in the MAR images, and the standard deviation remained lower for the MAR images at all distances from the insert. The artifact volume decreased using the novel MAR algorithm for all metal samples excluding aluminum (*p* < 0.001) and all hip prostheses (*p* < 0.05).

**Conclusion:**

Varian's HyperSight MAR reconstruction algorithm shows a reduction in metal artifact metrics, motivating the use of MAR reconstruction for patients with metal implants.

## 1 INTRODUCTION

Radiotherapy treatment planning requires accurate imaging of patient anatomy to identify and contour targeted structures and surrounding healthy tissues. Typically, Hounsfield unit (HU) values in the computed tomography (CT) images are converted to electron densities used in the calculation of radiation dose. Metallic implants, such as dental fillings, wires, screws, spine implants, joint prostheses, surgical clips, coils, and cardiac electronic devices, can induce metal artifacts and alter densities during the CT reconstruction, obscuring surrounding anatomy.^1^, ^2^, ^3^ Low‐energy photons from the imaging spectra have a higher probability of attenuation by these materials due to the atomic number dependence of the photoelectric effect. The assumption of monochromatic x‐rays in the reconstruction of a hardened beam may produce shading, streaking or cupping artifacts. The direction of particle propagation is also altered at the implant from Compton scattering, which increases with higher physical and electron density materials. Reconstruction algorithms assume x‐rays travel in a straight line from tube to detector, resulting in incorrect estimation of absorbed photons from these effects. This distorts the image producing dark streaks along the axis of high attenuation, surrounded by bright streaks.^4^ The extent of metal artifacts primarily depends on the implant's composition (atomic number and density), size, and shape, in addition to its orientation with respect to the imaging plane.^5^ The deteriorated image quality and incorrect HU can lead to inaccurate tumor/organ delineation and dose miscalculations, possibly resulting in underdosing of the tumor and overdosing of surrounding organs.^6^, ^7^ Furthermore, artifacts present in CBCT images acquired during treatment can affect contours of the influencer structures used to guide the propagation of the target volume from the planning CT in adaptive radiotherapy, and also cause difficulties in daily patient positioning and quantifying setup uncertainty. The backscatter of particles from the treatment beam at the high‐Z implant can also alter the dose distribution in that area and lead to hotspots that are not predicted by convolution superposition dose calculation algorithms.^8^, ^9^, ^10^ This further highlights the need for accurate visualization of both the implant itself and surrounding anatomy.

Several studies have demonstrated improvement in contrast‐to‐noise ratio, soft tissue delineation, Hounsfield Unit (HU) uniformity, and the reduction of streak artifacts and image noise with iterative CBCT (iCBCT) reconstruction relative to filtered back‐projection (FBP).^11^, ^12^, ^13^ Varian's iCBCT with Acuros (iCBCT Acuros) reconstruction mode includes a scatter correction^14^ and is recommended by the manufacturer for highest accuracy of HU units. The new advanced radiation therapy imaging platform, HyperSight,^15^ is equipped with a large CBCT imager (86 cm x 43 cm), reduced acquisition time (6 s), and offers image reconstruction advancements, including Feldkamp Davis Kress (FDK) back‐projection, iCBCT, iCBCT Acuros, and iCBCT metal artifact reduction (MAR). Preliminary analyses have shown that reconstructed CBCTs acquired by HyperSight using iCBCT Acuros displayed image quality and Hounsfield unit accuracy comparable to standard fan‐beam CT.^16^

MAR reconstruction algorithms aim to enhance image quality and reduce distortion in CT scans of patients with metal prostheses or implants. Iterative reconstruction is an advanced technique that reduces metal artifacts and improves image quality by performing a series of repeated reconstructions with newly derived adjustments being applied at each iteration, while taking into account physical data and photon statistics to reduce the metal artifact.^5^ Several investigations compared MAR algorithms to standard FPB methods for reconstructing CT images with metal implants, showing significantly improved CT image quality for large metal implants, but noted that the MAR algorithm introduced blurring artifacts and reduced the image quality with small metal implants.^17^, ^18^, ^19^

The MAR algorithm implemented in HyperSight does not require a priori knowledge of the type of object causing the artifact, for example, hip prosthesis. The proposed algorithm is similar to the standard iCBCT Acuros algorithm, but it performs an advanced inpainting in projection space, which aims at maximizing both inter and intra‐projection consistency of inpainted regions. This method requires different parametrizations depending on the type of metals present inside the scanned body. Therefore, as a first step of the MAR algorithm, a classification of the actual metal burden in the scan is performed. This classification is based on topological analysis applied to initial iterative reconstruction. The MAR algorithm only operates on metal burdens that fall within the body. Currently, the classification accounts for dental metals, orthopedic prostheses, and fiducials. In the case when the classification is unable to determine one of these types, a fallback parametrization is selected. Further details on the reconstruction algorithm are proprietary to Varian and omitted from this article.

This article presents the first comprehensive report on the effectiveness of the novel MAR reconstruction method. The purpose of this work is to characterize the MAR algorithm for reconstruction of Varian HyperSight CBCT images and compare improvements in image quality with the standard iCBCT Acuros reconstruction for a range of potentially artifact‐generating metals in radiotherapy patients.

## 2 METHODS AND MATERIALS

Three phantoms were used to simulate patients with high density implants and produce representative metal artifacts in CBCT images. Two phantoms were designed in‐house (using a solid water block and water tank background) and one was a commercial product (Advanced RED Phantom, Sun Nuclear). Each phantom was composed of a variety of high‐Z implants, surrounded by a tissue HU‐equivalent background. All CBCTs were acquired with the HyperSight imaging system, IGRT mode, using the preset Pelvis protocol (125 kV, 469 mAs) to allow a large imaging field. Two images were acquired for each setup; the first was reconstructed using iCBCT Acuros, and the second was reconstructed using iCBCT Acuros with MAR. A reference CBCT of each phantom was acquired with no metal insert to obtain baseline metrics for both reconstruction modes.

### 2.1 Experimental setup: Solid water block phantom

A 30 cm (length) x 30 cm (width) x 1 cm (depth) solid water block was fitted with a central cavity measuring 5.5 cm x 5.5 cm x 0.5 cm. Inserts of copper, aluminum, stainless steel, and titanium (5.5 cm x 5.5 cm x 1 ‐ 5 mm in thickness) and sample dental implant materials (Amalgam, Au, Co‐Cr, and Ti 6Al‐Yv) were sequentially used to fill the cavity. Two solid water blocks, each measuring 30 cm x 30 cm x 5 cm, were positioned above and below the solid water block containing the samples. CBCT images of the solid water block phantom were acquired and reconstructed using MAR and iCBCT Acuros. The cavity was then filled with solid water, and reference iCBCT Acuros and MAR‐reconstructed images of the phantom were acquired.

### 2.2 Quantitative analysis: Solid water block phantom

The signal‐to‐noise ratio (SNR) and artifact index (AI), defined in Equations 1 and 2, respectively, were measured in a 3 mm thick spherical shell‐structure volume of interest (VOI) with a radius of 30 mm centered around the cavity, and compared to baseline VOI metrics from the solid water‐only reference images.

(1) $$\mathrm{SNR}\;=\;\frac{\mathrm{Mean}\left(\text{VOI}\right)}{\text{SD}\left({\text{VOI}}_{\mathrm{background}}\right)}$$

(2) $$\mathrm{AI}\;=\sqrt{\text{SD}{\left(\text{VOI}\right)}^{2}-\text{SD}{\left({\text{VOI}}_{\mathrm{background}}\right)}^{2}}$$

To ensure no metal artifact was captured in the background volume, $VOI_{\mathrm{background}}$ was defined as the same spherical shell‐structure in the solid water‐only reference images. These two metrics relate to different artifact characteristics; SNR is effective in characterizing an artifact with uniform HU values (i.e., shadowing), while AI would indicate large variations in HU (i.e., streaking).

The structural similarity index measure (SSIM), peak signal‐to‐noise ratio (PSNR), and mean‐square error (MSE) values were computed using the solid water‐only phantom images as the reference. Pixels outside of the phantom volume and within the insert cavity were excluded from the analysis. SSIM is expressed as:

(3) $$\mathrm{SSIM}\;\left(x,y\right)=\frac{\left(2\mu_{x}\mu_{y}+C_{1}\right)\left(2\sigma_{xy}+C_{2}\right)}{\left(\mu_{x}^{2}+\mu_{y}^{2}+C_{1}\right)\left(\sigma_{x}^{2}+\sigma_{y}^{2}+C_{1}\right)}\;$$

where μ_x_, μ_y_, σ_x_,σ_y_, and σ_xy_ are the means, standard deviations, and cross‐covariance for the image x and reference image y. By default, the SSIM function uses regularization constants C_1_ = (0.01*L)^2^ and C_2_ = (0.03*L)^2^ where L is the dynamic range among both images. PSNR is defined as:

(4) $$\mathrm{PSNR}\;=\frac{10*\log_{10}\;\left({\text{peakval}}^{2}\right)}{\text{MSE}}\;$$

where peakval is the maximum pixel value in the reference image data, and MSE is defined as:

(5) $$\mathrm{MSE}\;=\frac{1}{MN}\;\sum_{i\;=\;0}^{M-1}\sum_{j\;=\;0}^{N-1}{\left(x\left(i,j\right)-y\left(i,j\right)\right)}^{2}$$

Here, *M* and *N* are the dimensions of the images x and y.

**FIGURE 1:**
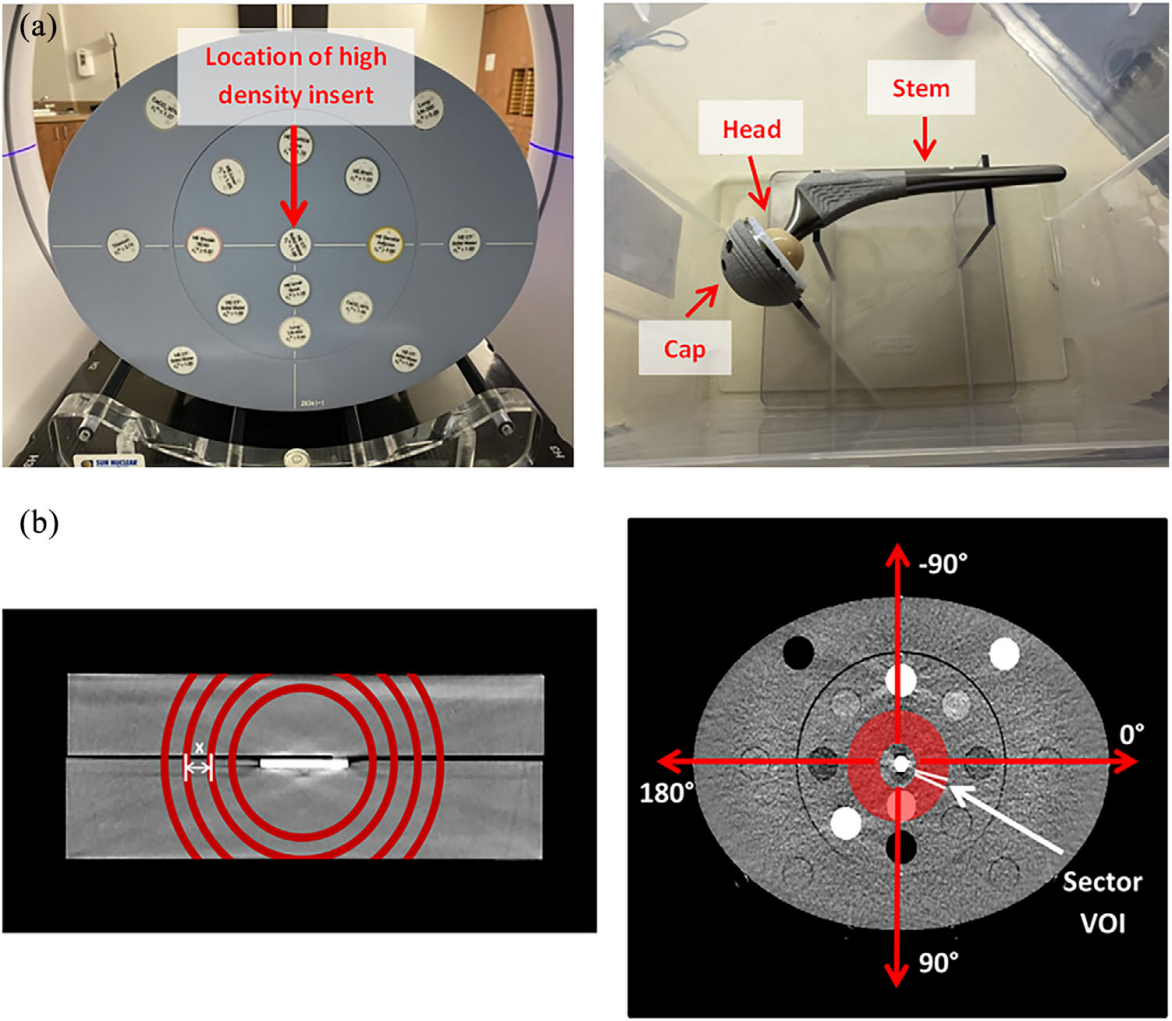
(a) The AED phantom (left), and the empty water tank phantom with an Omnifit EON stem (right), and (b) the depiction of the solid water block phantom with spherical shell VOIs of increasing radius (left) and the AED phantom with a sectored VOI, not to scale (right).

To evaluate the mean and standard deviation of HU as a function of distance from the center of the inserts, the radius of the spherical shell‐structure was increased from 30 to 70 mm in increments of 10 mm, shown in Figure 1b, and the mean and standard deviation of HU within the structure were recorded and analyzed.

The artifact volume of the phantom (excluding the cavity) was estimated by calculating the Second and 98th percentiles of HU in the CBCT of the phantom with the solid water alone, then summing the volume of all voxels with HU values outside this range in the CBCTs with metal inserts.

The *p*‐values for SNR, AI, SSIM, PSNR, MSE, and the artifact volume were computed from a paired‐sample *t*‐test with a null hypothesis being that the difference in MAR and iCBCT Acuros values comes from a normal distribution with mean equal to zero, at a 5% significance level.

### 2.3 Experimental setup: Advanced electron density phantom

The advanced electron density (AED) phantom^20^ is a thorax‐shaped phantom, 40.0 cm x 30.0 cm 26.5 cm in size. It is comprised of water‐equivalent material, and accommodates 16 rods that mimic a wide range of human tissues (i.e., cortical bone, inner bone, lung, and liver). The phantom was setup using 12 rods of various tissue‐equivalent densities and three rods of pure solid water. Three high‐density rods of aluminum, stainless steel, and titanium were sequentially placed in the center. The AED phantom and rod arrangement are shown in Figure 1a. iCBCT Acuros and MAR‐reconstructed CBCTs of the phantom were acquired with each high‐density rod. A solid water rod was then placed in the central position to generate reference iCBCT Acuros and MAR‐reconstructed images with the same tissue HU‐equivalent background arrangement.

### 2.4 Quantitative analysis: Advanced electron density phantom

The SNR and AI metrics were measured in the nine closest rod VOIs surrounding the implant. Since each of the rod VOIs in the AED phantom has a different density quoted by the manufacturer, and hence HU value, the SNR of each one (except for solid water) is expected to be non‐zero. Therefore, the SNR differences between each rod VOI and corresponding VOI from the reference images were evaluated.

The artifact‐dependence on angle was tested by introducing a polar coordinate system and transforming the linear cartesian coordinates to the polar ones, including angle and radius. A hollow cylindrical VOI of radius 30–55 mm and height of 10 mm, centered on the high‐density insert, was divided into 72 evenly spaced sector VOIs (increments of 5 degrees). The angular orientation is defined by the arrows in Figure 1b. The mean HU value within each sector was calculated and subtracted from the corresponding baseline mean HU value from the reference image. The standard deviation of relative differences was used to quantify the extent of streaking in each image.

### 2.5 Experimental setup: Hip prosthesis water tank phantom

Four Stryker orthopedic hip prostheses consisting of a Trident 1 tritanium cup (titanium), Alumina ceramic head (high‐purity Al_2_O_3_), and one of four stems: Omnifit EON (Forged CoCr), Accolade II (titanium Ti‐6Al‐4 V ELI), Securfit Max (titanium Ti‐6Al‐4 V ELI), or Exeter (Orthinox Stainless Steel Rex 734)^21^ were secured on a thin Lexan stand attached to the bottom of a (26 cm x 37 cm x 23 cm) container filled with water. This setup aimed to mimic the approximate location and direction of a hip implant within a patient simulated in the head‐first supine position. The hip prosthesis was positioned centrally in the transversal plane to allow an analysis of the artifact surrounding all sides of the prosthesis. iCBCT Acuros and MAR‐reconstructed images of the prostheses with each stem‐type were acquired, and all components of the prostheses were removed from the stand to acquire the reference image. The labeled hip prosthesis is shown in Figure 1a.

### 2.6 Quantitative analysis: Hip prosthesis water tank phantom

A generalized volume was contoured as the outline of the hip prosthesis, copied to all CBCT images. The same volume was used for all setups, and was inclusive of all parts of the implant, in all four cases. This volume was subtracted from the background VOI extending to the edges of the container. The SNR and AI metrics were computed from the resultant background VOI for both reconstruction modes and compared to baseline metrics from the same VOI in the reference images. Similar to the solid water block phantom, the artifact volume of the phantom background was estimated by excluding the same generalized hip prosthesis VOI from all images. To quantify the size of artifact present in each image, the second and 98th percentiles of HU in the CBCT of the phantom with no prosthesis were computed, and the volume of all voxels with HU values outside this range was summed for the phantoms with the hip prostheses.

The *p*‐values for SNR, AI, SSIM, PSNR, MSE, and the artifact volume were computed similarly as for the solid water phantom.

The naming convention for each insert‐type, as referred to in the figures, is summarized as follows:

(i) Square metal inserts: Al (aluminum), Cu (copper), SW (solid water), Steel (stainless steel), Ti (titanium); (ii) Dental implant pieces—Au (gold), Amal (Amalgam), Co‐Cr (cobalt‐chrome), Ti 6Al‐Yv (90% Titanium, 6% Aluminum, 4% Vanadium), No implant (solid water); (iii) AED metal rods: Al (aluminum), S Steel (stainless steel), Ti (titanium), SW (solid water); Hip prostheses: Omnifit (forged cobalt‐chrome), Accolade II (90% Titanium, 6% Aluminum, 4% Vanadium), Securfit Max (90% Titanium, 6% Aluminum, 4% Vanadium), Exeter (stainless steel).

## 3 RESULTS

Figure 2 shows selected images acquired for (a) the solid water block with amalgam and Co‐Cr dental implants, (b) the AED phantom with a titanium rod in the central insert, and (c) the water tank phantom with an Exeter (stainless steel) hip implant. The window level was fixed for all images (−175 to 200 HU).

**FIGURE 2:**
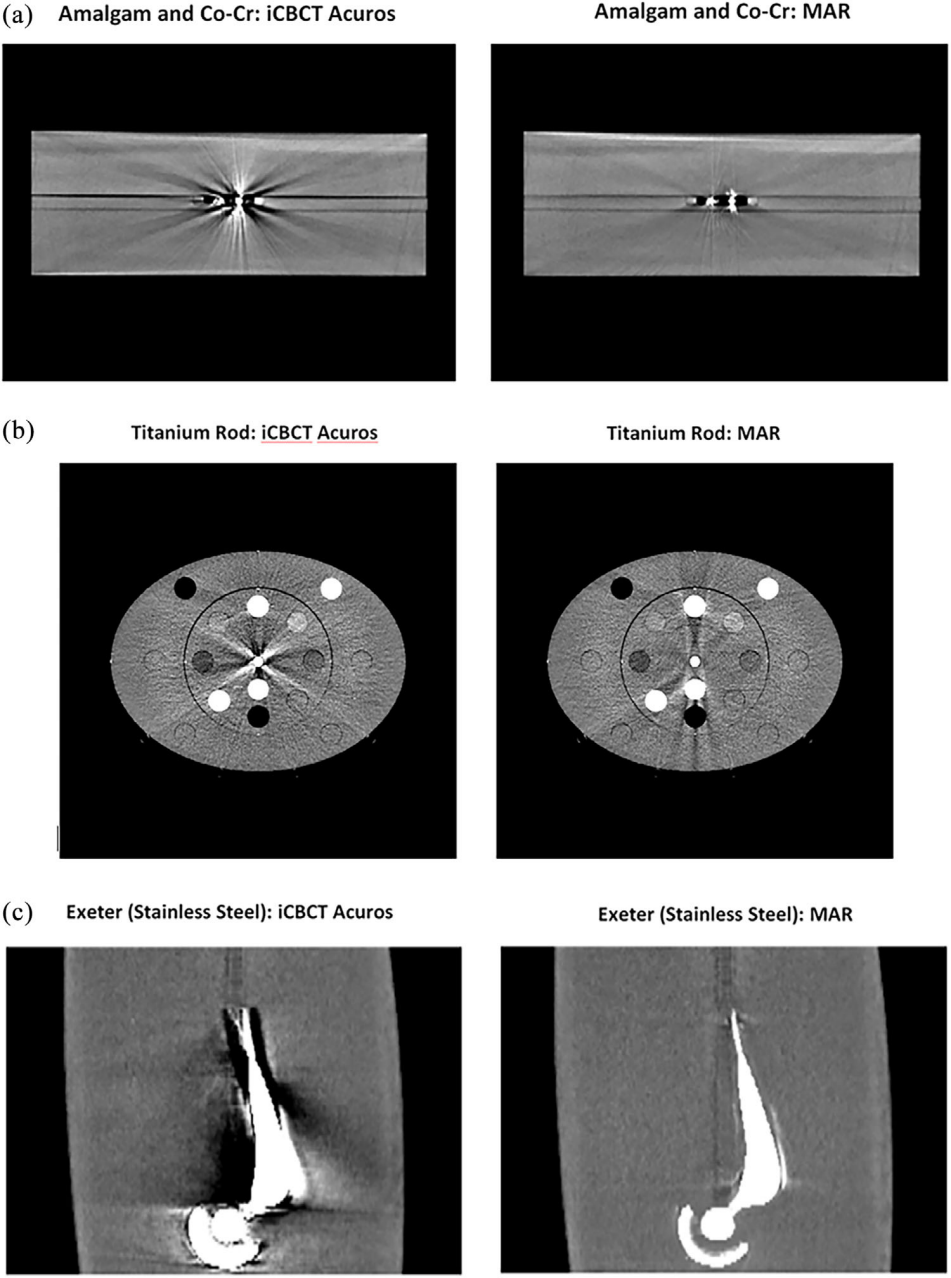
iCBCT Acuros and MAR‐reconstructed HyperSight CBCTs for (a) the solid water block with amalgam and Co‐Cr dental implants, (b) the AED phantom with a titanium rod in the central insert, and (c) the water tank phantom with an Exeter (stainless steel) hip implant. The window level was fixed for all images (−175 to 200 HU).

**FIGURE 3:**
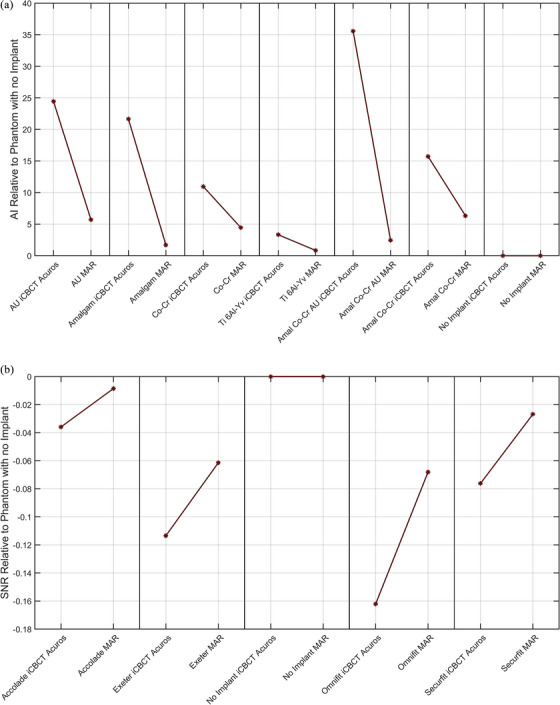
Selected VOI metrics computed from AXB and MAR Hypersight CBCTs of (a) the dental implant solid water block phantom and (b) the prosthetic water tank phantom. The quantities are subtracted from those computed on the phantom image with no metal artifacts.

Figure 3 displays the following image quality metrics computed in the VOI for the AXB and MAR CBCTs: (a) AI from the dental implant solid water block phantom, (b) SNR from the prosthetic water tank phantom. The AI metric from the dental implant phantom is presented here as an example since a streaking artifact was clearly observed, while the SNR metric from the hip prostheses phantom is shown because shadowing was the predominant effect.

The SNR and AI values for each reconstruction mode were computed for the background VOI in the solid water block phantoms and water tank phantom. A decrease in SNR, AI, and MSE, and an increase in SSIM and PSNR was observed in MAR‐reconstructed images for all metal implant‐types except for the Al square insert. For the square metal insert phantom, the *p*‐values computed from the MAR and iCBCT Acuros grouped data were *p*_SNR_ = 0.002, *p*_AI_ = 0.001, *p*_SSIM_ = 0.0003, *p*_PSNR_ = 0.001, and *p*_MSE_ = 0.004. For the dental implant phantom, the *p*‐values were *p*_SNR_ = 0.226, *p*_AI_ = 0.022, *p*_SSIM_ = 0.00005, *p*_PSNR_ = 0.003, and *p*_MSE_ = 0.0002, and for the hip prosthesis phantom, *p*_SNR _= 0.027, *p*_AI _= 0.006, *p*_SSIM_ = 0.02, *p*_PSNR_ = 0.04, and *p*_MSE_ = 0.003. A *p*‐value below 0.05 indicates that the MAR and iCBCT Acuros metrics are significantly different from each other.

The relative mean, SNR, and AI values for each reconstruction mode were computed for various tissue‐equivalent rod VOIs in the AED phantom. The SNR values for two, four, and six (out of the nine) tissue‐equivalent rod VOIs were closer to the corresponding SNR values from the reference image for the aluminum, stainless steel, and titanium rod inserts, respectively. The AI values for six, seven, and seven (out of the nine) tissue‐equivalent rod VOIs were closer to the corresponding AI values from the reference image for the aluminum, stainless steel, and titanium rod inserts, respectively.

**FIGURE 4:**
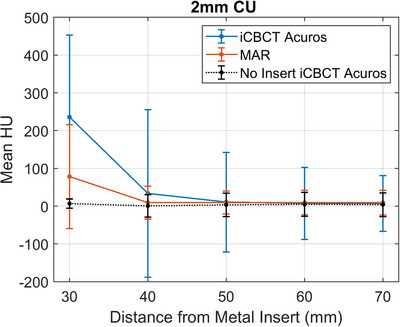
Mean HU with increasing distance from the center of the 2 mm CU square insert in the solid water block phantom, as an example, for MAR and iCBCT Acuros reconstructed images. The error bars indicate the HU standard deviation within the VOI. The data for the reference image (no metal insert reconstructed with iCBCT Acuros) is plotted to outline the ground truth.

The mean HU and standard deviation with increasing radius of the shell‐structure is shown in Figure 4 for 2 mm Cu as a representative example.

The standard deviation of the 30 mm radius spherical shell in the reference phantom image (with no metal insert) was 12.06 HU. The mean HU was within one standard deviation of zero (the expected HU of the solid water background) within 40 mm from the center for all metal inserts in the MAR‐reconstructed images and only in the 3 mm aluminum and 1 mm titanium square insert iCBCT Acuros reconstructed images. The standard deviation remained lower for the MAR‐reconstructed images at all distances from the insert for all square metal inserts except for aluminum.

**FIGURE 5:**
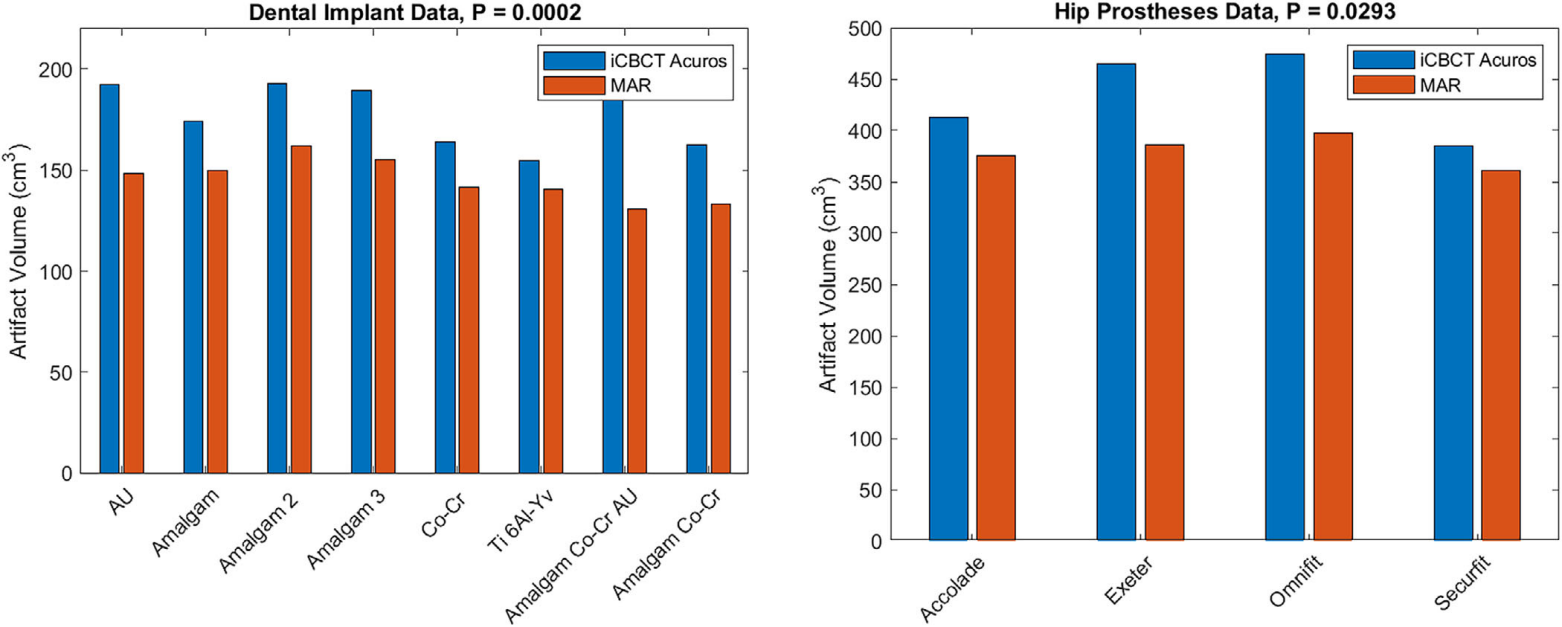
Comparison of artifact volumes obtained from HyperSight iCBCT Acuros and MAR‐reconstructed images of the dental implant and hip prosthesis phantoms. The *p*‐values shown in the title are computed for the iCBCT Acuros and MAR artifact volumes using a paired‐sample *t*‐test.

The artifact volumes are presented in a bar graph for the dental implant and hip prosthesis phantoms in Figure 5.

The *p*‐values for the iCBCT Acuros and MAR artifact volumes are computed from a paired‐sample *t*‐test. The artifact volume was significantly lower for the MAR‐reconstructed images of metal inserts excluding aluminum (*p* = 0.0013), all dental implants (*p* = 0.0002), and all hip prostheses (*p* = 0.006).

**FIGURE 6:**
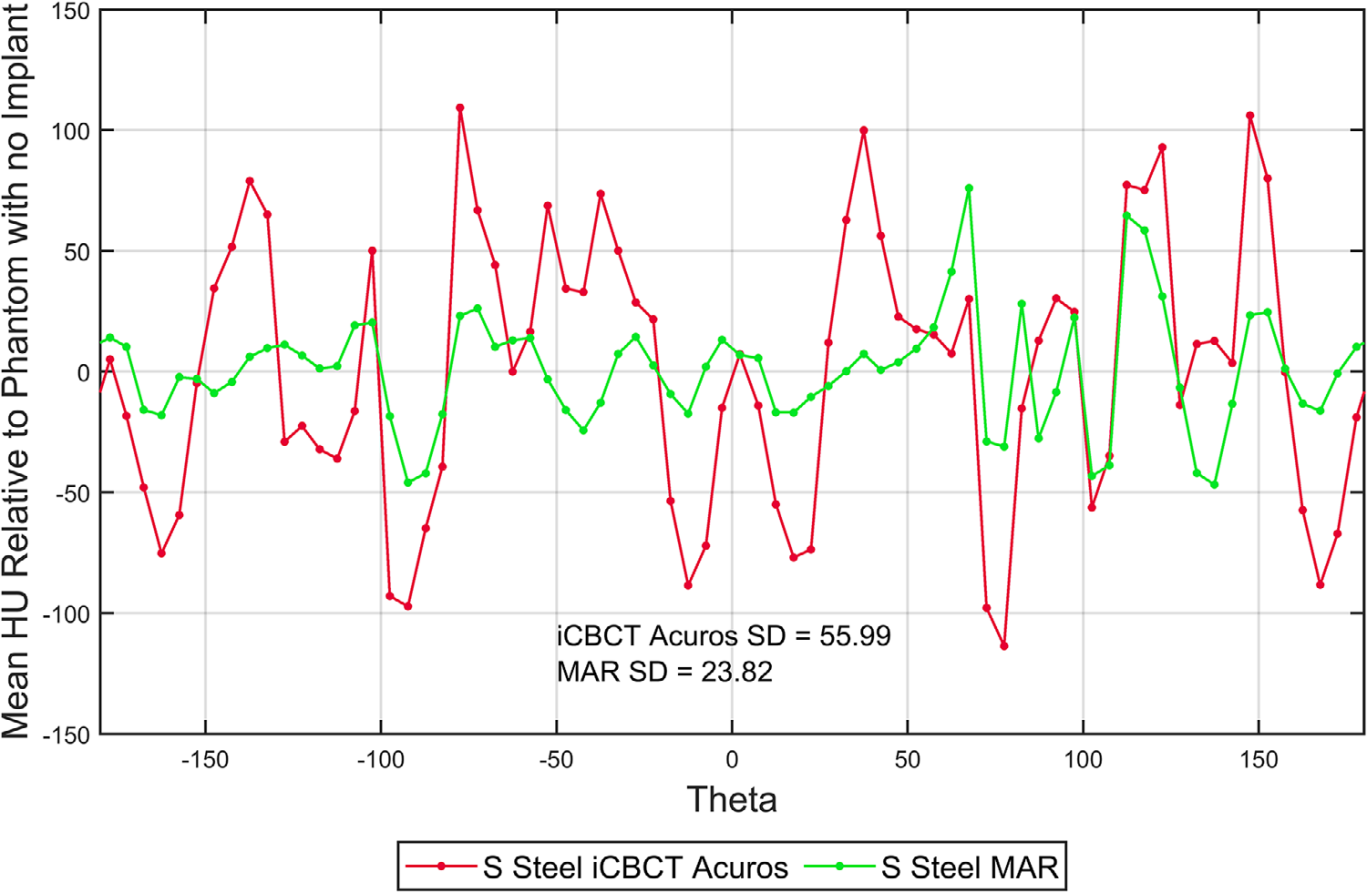
The mean HU differences relative to the phantom with no insert of 72 evenly spaced sectors around metal implant as a function of sector angle for the AED phantom with a stainless steel insert.

The CBCT images of the AED phantom appeared to show a consistent pattern of image artifact (streaking) with respect to angle. The artifact dependence on angle is shown in Figure 6a, where the mean HU of the 72 evenly spaced sectors around metal implant are plotted as a function of sector angle for the AED phantom with stainless steel as an example. Figure 6 shows the corresponding mean HU differences relative to the reference image with no metal rod implant.

## 4 DISCUSSION

Metallic implants commonly produce CT artifacts that may compromise image quality and limit the visibility of the target and adjacent organs. In this investigation, a series of images of phantoms with different metal implants were reconstructed with Varian's HyperSight MAR algorithm, and compared to the same projections reconstructed using iCBCT Acuros. There was a reduction of metal artifacts observed in the CBCT images among all metal insert‐types used in each phantom except for the aluminum square insert and the aluminum rod. The implementation of MAR would particularly be beneficial in cases where the target and important organs are located near an implant. For example, it would allow for more accurate visibility and contouring of organs near the hip for prostate treatments, or near the oral cavity where dental fillings may be present for head and neck treatments. A baseline case without artifacts was established for each phantom by acquiring images in the absence of an implant. The improved HU relative to nominal, as observed from our data when using MAR, would lead to less uncertainty in the dose calculation in and near any high density.

The SNR of a VOI in a uniform water‐equivalent background with no artifacts is expected to be close to zero. The difference in standard deviation between any VOI and the background should also be close to zero when there is no artifact, which would result in an AI close to zero. The SSIM, PSNR, and MSE metrics evaluate the quality of an image by comparing it to a reference image that serves as the ground truth. An SSIM value close to one, a higher PSNR, or a lower MSE value indicates that the image in question is similar to the reference, signifying better image quality. The SNR and AI image quality metrics extracted from the chosen VOIs in the solid water block phantom and hip prosthesis phantom were significantly closer to baseline except for the SNR from the dental samples (*p*_SNR_ = 0.226) when using MAR compared to iCBCT Acuros. The artifact produced by the dental implants may have not been strong enough to detect a significant difference between reconstruction modes, as seen with the other phantoms. Furthermore, the dental implants produced exhibited a prominent streaking artifact as opposed to shadowing, which is more effectively detected by AI than SNR. The SSIM, PSNR, and MSE, computed using image data of the entire phantom, excluding the implant region, showed significant improvement in image quality (i.e., higher SSIM and PSNR, and lower MSE) for the MAR‐reconstructed images compared to iCBCT Acuros.

The aluminum implants demonstrated a larger disagreement from baseline for MAR images than iCBCT Acuros among all image quality metrics. These findings align with the results from similar studies on MAR for CT—the application of MAR methods in CT scans can significantly improve image quality for patients with metal; however, MAR introduces limitations such as other artifacts or image quality degradation from blurring with small or less dense metal implants.^17^, ^18^, ^19^ Aluminum alone is not common in medical implants, or as dense as the other materials investigated in this study, so it may have not exceeded the thresholding used to identify artifact‐inducing metal objects in the MAR reconstruction algorithm. However, it is used as an alloying element, for example, the Accolade II and Securfit Max hip implants (composed of 90% titanium, 6% aluminum, and 4% vanadium), for which an improvement with MAR was achieved. Our results showed superior quality in iCBCT Acuros‐reconstructed images for the phantoms with aluminum‐alone implants, so MAR should only be used for patients with implants of known material that produce observable metal artifacts, as it may introduce new image artifacts for lower density materials.

Out of the 18 tissue‐equivalent rod VOIs evaluated for the stainless steel and titanium inserts in the AED phantom (collectively), there was a reduction of SNR values for 10 rods (56%), and a reduction of AI values for 14 rods (78%), relative to the phantom with no metal insert. It is possible that the remaining rods did not show a reduction in artifact metrics due to the implementation of the classification scheme, which first determines which anatomical region the scan is within, head or body, and the AED phantom (and other phantoms tested in the study) may not fall into either of these categories. For this reason, a more anatomically accurate phantom might show greater improvement in metrics within various tissue‐equivalent VOIs than the tested phantoms.

The artifact volume, defined as the volume of pixels outside of the implant exceeding the second and 98th percentiles of HU in the CBCT of the phantom with the solid water alone, significantly decreased when using the novel MAR algorithm for all metal inserts excluding aluminum (*p* = 0.0013), all dental implants (*p* = 0.002), and all hip prostheses (*p* = 0.006). MAR was most impactful at distances closer to the metal, which could be advantageous for patients who have critical anatomy close to an implant.

The high/low HU values at different angles in Figure 6 indicate bright/dark streaks surrounding the insert, respectively. The standard deviation of relative differences across a range of angles in the AED phantom was lower for the MAR image with the stainless steel insert by 57%, and with the titanium insert by 31%, reflecting the reduction of artifact compared to iCBCT Acuros; however, this was not the case for the aluminum rod, which had an increase in standard deviation of 125%. The larger mean HU differences (from reference) at +90 and −90 degrees in Figure 6 could be related to the high‐density tissue VOIs along that path. Streaking artifacts commonly occur along the tangent line to boundary of two or more high density objects.^22^ When a high‐density tissue‐equivalent volume was introduced (i.e., bone the in the AED phantom), heavier streaking along the path to the metal rod was more apparent in the MAR‐reconstructed images. It could be important to take into account neighboring high‐density anatomy in the patient, since the orientation of maximum artifact may be affected by it.

A limitation of this study is that the distance between high‐density tissue rods and the metal rod could not be altered, as the rod insert positions are fixed in the AED phantom. A more extensive evaluation of the effect of metal artifact on dependence on position and density of neighboring structures relative to the implant could be conducted in future studies. Additionally, the central position of inserts across all phantoms allowed for symmetry in the analysis of the artifact surrounding the insert; however, we did not explore the dependence of insert position relative to the phantom and encourage it as a continuation of the study. As a first step in our study, we used a simple design of phantoms to get a more accurate assessment of the artifact produced by an isolated feature, but the MAR algorithm classification scheme may have been bypassed in some cases due to the flat phantom geometry, and some results could be better in clinical anatomy. A more complex design of the experiment which would study the interaction of artifacts with realistic anatomy, or the joint effect of multiple implants is a feasible continuation of the study. The work presented here focuses on metal artifacts observed in cases of standard radiotherapy. Incorporating simulations of high‐dose rate (HDR) brachytherapy, for example, with gold fiducial markers, HDR prostate needles, and HDR gynecological applicators could be considered as an avenue for future experiments. The inclusion of real patient data and an exploration of additional factors, such as the assessment of organs at risk and the dosimetric evaluation of realistic target volumes, will be important for translating our findings into practical applications within a clinical setting.

## 5 CONCLUSION

HyperSight offers a new MAR image reconstruction method for patients whose radiation treatment may be negatively impacted by the effects of metal artifacts. The analysis conducted in this study confirmed a significant improvement in image quality and lower sensitivity to metal artifacts for all metal samples except for the aluminum inserts. These results provide firm support for the use of MAR reconstruction in a clinical setting for patients with metal implants, and highlight advantages of the new HyperSight system for more accurate imaging and targeted treatment. Future work will expand on the validation of MAR on other implant types and clinically relevant backgrounds.

## AUTHOR CONTRIBUTIONS

Abby Yashayaeva, Robert Lee MacDonald, and Amanda Cherpak contributed to the design of the work, data acquisition, and all authors contributed to the analysis and interpretation of data. Abby Yashayaeva drafted the manuscript and all authors critically reviewed and revised it. All authors approved the final version to be published and agree to be accountable for all aspects of the work in ensuring that questions related to the accuracy or integrity of any part of the work are appropriately investigated and resolved.

## CONFLICT OF INTEREST STATEMENT

Abby Yashayaeva has no conflicts of interest to declare. Amanda Cherpak, Lee MacDonald, and James Robar are members of an advisory group with Varian.

## Data Availability

The data that support the findings of this study are available from the corresponding author upon reasonable request.
